# Association of emergency intensive care unit occupancy due to brain-dead organ donors with ambulance diversion

**DOI:** 10.1038/s41598-025-97198-7

**Published:** 2025-04-12

**Authors:** Tetsuya Yumoto, Takafumi Obara, Takashi Hongo, Tsuyoshi Nojima, Kohei Tsukahara, Masaki Hisamura, Atsunori Nakao, Takashi Yorifuji, Hiromichi Naito

**Affiliations:** 1 Department of Emergency, Critical Care, and Disaster Medicine, Faculty of Medicine, Dentistry, and Pharmaceutical Sciences, Okayama University, 2-5-1 Shikata-cho, Kita-ku, Okayama, 700-8558 Japan; 2 Department of Epidemiology, Faculty of Medicine, Dentistry and Pharmaceutical Sciences, Okayama University, 2-5-1 Shikata-cho, Kita-ku, Okayama, 700-8558 Japan

**Keywords:** Ambulance diversion, Bed occupancy, Brain death, Emergency medical services, Intensive care units, Organ donation, Epidemiology, Epidemiology

## Abstract

Our study aims to explore how intensive care unit (ICU) occupancy by brain-dead organ donors affects emergency ambulance diversions. In this retrospective, single-center study at an emergency ICU (EICU), brain-dead organ donors were managed until organ procurement. We classified each day between August 1, 2021, and July 31, 2023, as either an exposure day (any day with a brain-dead organ donor in the EICU from admission to organ procurement) or a control day (all other days). The study compared these days and used multiple logistic regression analysis to assess the impact of EICU occupancy by brain-dead organ donors on ambulance diversions. Over two years, 6,058 emergency patients were transported by ambulance, with 1327 admitted to the EICU, including 13 brain-dead organ donors. Brain-dead donors had longer EICU stays (17 vs. 2 days, *P* < 0.001). With 168 exposure and 562 control days, EICU occupancy was higher on exposure days (75% vs. 67%, *P* = 0.003), leading to more ambulance diversions. Logistic regression showed exposure days significantly increased ambulance diversions, with an odds ratio of 1.79 (95% CIs 1.10–2.88). This study shows that managing brain-dead organ donors in the EICU leads to longer stays and higher occupancy, resulting in more frequent ambulance diversions. These findings highlight the critical need for policies that optimize ICU resource allocation while maintaining the infrastructure necessary to support organ donation programs and ensuring continued care for brain-dead donors, who play an essential role in addressing the organ shortage crisis.

## Introduction

High-quality care of brain-dead donors in the intensive care unit (ICU) is key to increasing organ donation, honoring patients’ and families’ wishes, and improving transplant outcomes^1,1,1^.

ICU stays for brain-dead donors vary depending on specific causes and case details. In Spain, 50% of brain-dead donors spend less than 24 h in the ICU; however, stays may exceed this duration for those with less severe initial brain injuries or higher Glasgow Coma Scale scores^4^. In contrast, the mean ICU length of stay for brain death patients in the United States was reported to be 4 days^5^. In Japan, despite the absence of specific data, evidence from studies on end-of-life care suggests brain-dead donors often experience longer ICU stays compared to other patients^6,6,6,6^. Additionally, a recent study reported that brain-dead donors who experienced an episode of cardiac arrest had a median ICU stay of 9 days from admission to organ procurement^10^. These assumptions highlight ethical dilemmas in ICU bed allocation, comparing the care of severely ill patients with minimal survival odds to that of potential organ donors, whose management may offer significant societal benefits^11^. In fact, high ICU occupancy can lead to ambulance diversions and change ICU admission decisions for critically ill patients.^12,12^. Ambulance diversions often result in prolonged prehospital times, delays in care, and increased mortality among critically ill patients^14^.

The aging population and more ambulance calls have made ambulance diversion due to bed shortages a significant social issue^15^. Organ donation adds complexity to this issue, with increasing transplant demand in regions including the United States and Europe contributing to organ shortages^16^. Furthermore, the screening for brain death, particularly after cardiac arrest, is increasingly suggested before deciding on the withdrawal of life support^17,17^. Given these trends, studying how ICU occupancy by brain-dead donors affects ambulance diversions is vital, yet Japan lacks data on donors’ ICU stay lengths.

Our study, therefore, aims to explore the relationship between ICU occupancy by brain-dead organ donors and emergency ambulance diversions.

## Methods

The present study was approved by the Okayama University Hospital Ethics Committee (K2403-004) and was conducted in accordance with the declaration of Helsinki. ﻿ The ethics committee waived the need for written informed consent due to the retrospective nature of the study and the anonymity of the data.

### Study design and setting

This retrospective, single-center study was conducted at the Okayama University Advanced Critical Care and Emergency Medical Center from August 1, 2021, to July 31, 2023. The hospital has 12 Emergency ICU (EICU) beds, 2 of which are designated for SARS-CoV-2 (COVID-19) patients, managed by emergency and critical care physicians. The EICU accepts all critically ill or severely injured patients, including those with trauma, stroke, post-cardiac arrest, or sepsis. During the study period, 570 patients (43.3%) were transferred to a general ward, 468 (35.5%) were discharged to another hospital, 192 (14.6%) were discharged home, and 87 (6.6%) died in the ICU. Upon receiving a request from the scene, the attending emergency and critical care physician determines whether to accept a patient based on the potential severity of the patient’s condition, EICU occupancy, activity in the emergency department, and the availability of specialists, if needed. If a patient is not accepted, the reasons for rejection are recorded in the daily log chart as follows: EICU beds being occupied, ongoing care for severe patients, or the unavailability of specialists for specific treatments.

### Process of organ donation

Regarding the management of potential organ donors, patients with severe neurological damage are identified through neurological assessments and head computed tomography scans. The process of organ donation after brain death is detailed elsewhere^10^. Briefly, under the procedures of the Japan Organ Transplant Network, organ donation is generally proposed following the clinical confirmation of brain death. Clinical confirmation of brain death is based on the presence of deep coma, bilateral pupil dilation, absence of brain stem reflexes, and a flat EEG pattern. However, if a patient is identified as a potential organ donor due to devastating brain damage, the organ donation option may be presented prior to clinical confirmation of brain death. At our institution, after clinical confirmation of brain death, ad hoc multidisciplinary team meetings—held in addition to regular meetings—are organized to determine the appropriate timing and method for presenting organ donation information to the family^19^. During the study period, 19 out of 32 families (59.4%) who were presented with this information after the clinical confirmation of brain death of their family member declined to donate the patient’s organs. With family consent, the process necessitates two independent legal confirmations of brain death, conducted at a minimum interval of 6 h (or 24 h for children under 6 years old). These confirmations involve comprehensive neurological tests, including those mentioned above, along with an apnea test. Legal confirmation of brain death is performed by two designated specialists at the hospital, independent of the physician responsible for the patient. Patients remain in the EICU until this process is complete. In Japan, transferring potential donors between hospitals is tightly regulated.

### Regional emergency medical services system

Okayama Prefecture is split into four medical regions, with Okayama University Hospital serving the southeast, covering 5 cities and 0.92 million people over 1899 km^2^. Central dispatch centers in each city manage emergency responses. Okayama City, the largest and home to the hospital, has 0.72 million people in 789 km^2^. Ambulance crews assess and transport patients to appropriate hospitals. In Okayama City, a second tertiary hospital specializes in emergency and critical care, providing an additional resource for managing critically ill patients. However, when the EICU at Okayama University Hospital reaches high occupancy—particularly during the prolonged stays of brain-dead organ donors—we are forced to divert ambulances carrying patients who are critically ill or injured. This policy ensures patient safety while balancing resource limitations.

### Data collection

The daily log chart was thoroughly reviewed for the study period. The analysis specifically focused on recording the number of ambulance diversions that occurred, particularly those due to occupied EICU beds. Furthermore, we tracked the daily occupancy rate of EICU beds, including those designated for COVID-19 patients, each day at 10 a.m., along with the daily count of patients receiving invasive mechanical ventilation, continuous renal replacement therapy (CRRT), and extracorporeal membrane oxygenation (ECMO), monitoring device availability to gauge EICU patient severity and impact on emergency admission decisions.^20^. In addition, detailed information about patients who were admitted to the EICU and subsequently donated organs after brain death was retrieved from their medical records. This information was used to define the exposure day and the control day (as detailed below).

### Definition of exposure day and control day

To assess the impact of EICU bed occupancy by brain-dead organ donors, each of the 730 days from August 1, 2021, through July 31, 2023, was categorized as either an exposure day or a control day. An exposure day is defined as any day during which a brain-dead organ donor was present in the EICU from the time of admission until the day of organ procurement. Conversely, all other days within this timeframe were classified as control days.

### Outcome

The primary outcome was the number of ambulance diversions due to EICU bed occupancy, comparing days with brain-dead donor patients (exposure days) to days without them (control days). The secondary outcome was the ICU length of stay, which was also compared between exposure days and control days.

### Statistical analyses

Continuous data were reported as medians and interquartile ranges (IQR) or means and standard deviations, while categorical data were shown as frequencies and percentages. The Mann–Whitney U test and Chi-square test were used for comparing continuous and categorical variables, respectively. A multiple logistic regression was used to calculate adjusted odds ratios (OR) and 95% confidence intervals (CIs) for ambulance diversions associated with EICU occupancy, accounting for exposure/control days, the number of ambulance acceptances (as a proxy for emergency department crowding), the presence or absence of rejections due to specialist unavailability, COVID-19 occupancy, CRRT/ECMO operations, and temporal trends (per 6-month period over 2 years). The analysis aimed to evaluate the likelihood of ambulance diversions, with no diversions as the reference. Daily invasive ventilation operations were not adjusted for, considering their commonality in brain-dead donors and thus being an intermediate variable. As a sensitivity analysis, we used two alternative definitions of exposure day and control day. The first alternative defines an exposure day as any day on which multiple brain-dead organ donors were present in the EICU. The second alternative defines an exposure day as any day within the period from when a patient receives clinical confirmation of the state of brain death until the day of organ procurement. ﻿All tests were two-tailed, and a *P* value of < 0.05 was considered statistically significant. ﻿Analyses were conducted using Prism 10.0.3 (GraphPad, San Diego, CA) and IBM SPSS Statistics 26 (IBM SPSS, Chicago, IL).

## Results

During the 2-year study period, 6,058 emergency patients were transported by ambulance. Of these, 1327 patients were admitted to the EICU, with median age of 65 years (IQR, 44–78). Among these patients, 875 were men and 452 were women. Ninety-nine of the 1327 patients were under 18 years old.

### Comparison between exposure days and control days

**Fig. 1:**
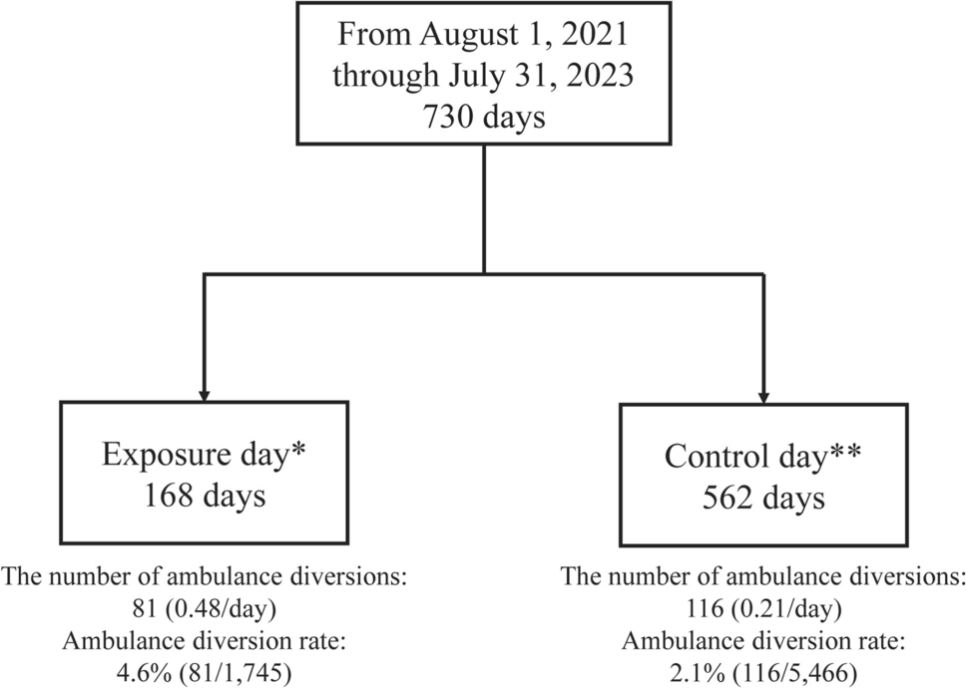
Flow of the study. *Exposure day is defined as any day within the 2-year period during which one or more of the 13 brain-dead organ donors were present in the EICU from the time of admission until the day of organ procurement. ** Control day is defined as any day within the 2-year period during which none of the 13 brain-dead organ donors were present, or when one or more of the 1,314 other patients (non-brain-dead organ donors) were present. The number of ambulance diversions and diversion rates were based on EICU occupancy.

**Fig. 2:**
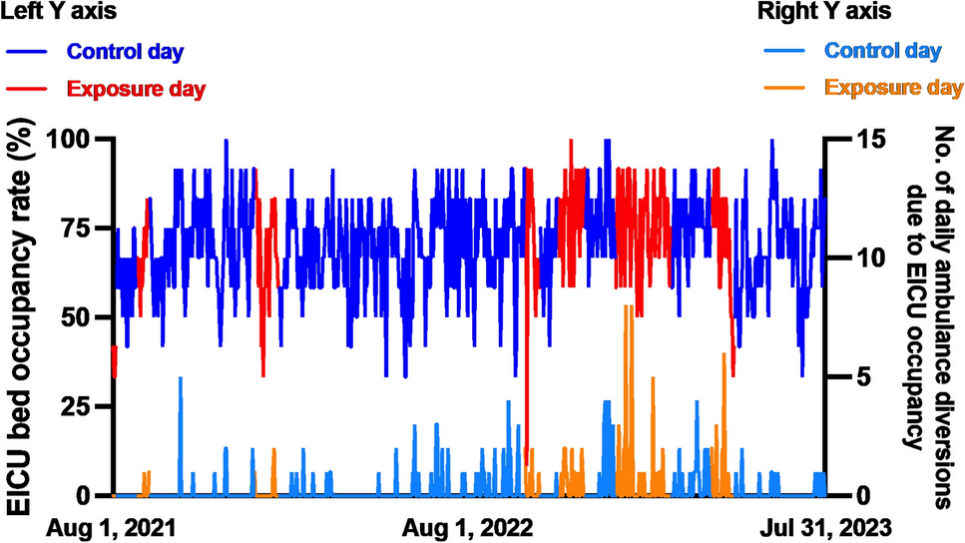
Trends in EICU bed occupancy rates and daily ambulance diversions attributed to EICU occupancy, contrasting exposure days with control days. EICU: emergency intensive care unit.

According to the definition of an exposure day and a control day, there were 168 days classified as exposure days and 562 days as control days. Figure 1 illustrates the flow of this study. Table 1 shows baseline characteristics of a comparison between exposure days and control days. EICU bed occupancy rates were higher on exposure days than control days (75% vs. 67%, *P* = 0.003). Number of daily ambulance diversions due to EICU occupancy were higher on exposure days than control days. Figure 2 illustrates trends in EICU occupancy and ambulance diversions by comparing exposure days to control days.

**Table 1:** A comparison between exposure days and control days.

	Exposure days n = 168	Control days n = 562	*P* value
Number of EICU beds occupied, median (IQR)^a^	9 (8, 10)	8 (7, 9)	0.003
Number of EICU beds occupied for COVID-19, median (IQR)^b^	1 (0, 2)	1 (0, 1)	0.444
EICU bed occupancy rate, median (IQR), %^c^	75 (67, 83)	67 (58, 75)	0.003
Daily count of patients receiving invasive mechanical ventilation			
Median (IQR)	5 (4, 6)	4 (3, 5)	< 0.001
Mean (SD)	4.9 (1.7)	4.0 (1.8)	
Daily count of patients receiving CRRT			
Median (IQR)	0 (0, 1)	0 (0, 1)	0.095
Mean (SD)	0.47 (0.68)	0.40 (0.69)	
Daily count of patients receiving ECMO			
Median (IQR)	0 (0, 0)	0 (0, 0)	0.548
Mean (SD)	0.07 (0.26)	0.06 (0.24)	
Number of daily ambulance acceptance cases, median (IQR)	8 (6, 11)	8 (6, 10)	0.307
Rate of ambulance acceptance, median (IQR), %	86 (75, 100)	88 (79, 100)	0.027
Number of daily ambulance diversions, n (%)			0.172
0	46 (27.4)	187 (33.3)	
1	45 (26.8)	161 (28.6)	
≥ 2	77 (45.8)	214 (38.1)	
Number of daily ambulance diversions due to EICU occupancy, n (%)			0.001
0	127 (75.6)	490 (87.2)	
1	22 (13.1)	45 (8.0)	
≥ 2	19 (11.3)	27 (4.8)	

^a^Maximum capacity of 12 beds.

^b^Maximum capacity of 2 beds.

^c^Calculated by dividing the number of EICU beds occupied by the total number of available EICU beds^12^, then multiplying by 100.

*IQR* interquartile ranges, *COVID-19* SARS-CoV-2, *SD* standard deviation, *CRRT* continuous renal replacement therapy, *ECMO* extracorporeal membrane oxygenation, *EICU* emergency intensive care unit.

### Comparison between brain-dead organ donors and other patients

Of the 1327 patients admitted, 13 donated their organs after being legally declared brain-dead. Of the 13 patients, the median time from admission to a diagnosis of brain death was 5 days (IQR, 4–7). The median time from a diagnosis of brain death to organ procurement was 12 days (IQR, 10–18). Supplemental Table 1 presents the baseline characteristics of the brain-dead organ donors and other patients. The length of ICU stay was significantly longer for brain-dead organ donors than for others (17 days vs. 2 days, *P* < 0.001).

### Multiple logistic regression analysis

A multiple logistic regression analysis revealed that exposure days were associated with an increase in ambulance diversions due to EICU occupancy (adjusted OR, 1.79; 95% CIs, 1.10–2.88), considering control days as the reference (Table 2).

**Table 2:** Adjusted effects of exposure days on daily ambulance diversions due to EICU occupancy, with exposure days defined as periods when brain-dead organ donors were in the EICU.

Variables	Adjusted OR	95% CIs
Exposure days	1.79	1.10–2.88
The number of ambulance acceptances	1.05	0.98–1.12
The presence or absence of rejections due to specialist unavailability	0.97	0.62–1.53
The number of COVID-19 beds occupied	1.18	0.88–1.57
Daily count of patients receiving CRRT	1.12	0.82–1.51
Daily count of patients receiving ECMO	1.15	0.42–2.18
Timeframe (per 3-month period)		
From August 1, 2021, to January 31, 2022	Reference	
From February 1, 2022, to July 31, 2022	1.65	0.76–3.67
From August 1, 2022, to January 31, 2023	2.97	1.41–6.55
From February 1, 2023, to July 31, 2023	2.56	1.27–5.43

*EICU* emergency intensive care unit, *OR* odds ratio, *CIs* confidence intervals, *COVID-19* SARS-CoV-2, *CRRT* continuous renal replacement therapy, *ECMO* extracorporeal membrane oxygenation.

### Sensitivity analysis

The first sensitivity analysis compared 55 exposure days (with multiple donors) to 675 control days, finding higher EICU occupancy on exposure days (75% vs. 67%, *P* = 0.003). A multiple logistic regression analysis indicated that exposure days were not significantly associated with increased ambulance diversions due to EICU occupancy (adjusted OR, 1.95; 95% CIs, 0.80–4.47) (Table 3).

**Table 3:** Adjusted effects of exposure days on daily ambulance diversions due to EICU occupancy, with exposure days defined by the presence of multiple simultaneous brain-dead organ donors.

Variables	Adjusted OR	95% CI
Exposure days	1.95	0.80 to 4.47
The number of ambulance acceptances	1.04	0.98 to 1.11
The presence or absence of rejections due to specialist unavailability	0.98	0.62 to 1.54
The number of COVID-19 beds occupied	1.18	0.88 to 1.57
Daily count of patients receiving CRRT	1.15	0.84 to 1.54
Daily count of patients receiving ECMO	0.92	0.39 to 2.02
Timeframe (per 3-month period)		
From August 1, 2021, to January 31, 2022	Reference	
From February 1, 2022, to July 31, 2022	1.41	0.66 to 3.09
From August 1, 2022, to January 31, 2023	3.25	1.56 to 7.10
From February 1, 2023, to July 31, 2023	2.39	1.17 to 5.11

*EICU* emergency intensive care unit, *OR* odds ratio, *CI* confidence intervals, *COVID-19* SARS-CoV-2, *CRRT* continuous renal replacement therapy, *ECMO* extracorporeal membrane oxygenation.

The second analysis compared 135 exposure days with donors to 595 control days, showing increased EICU occupancy on exposure days (75% vs. 67%, *P* = 0.031). Similarly, a multiple logistic regression analysis found no statistically significant association between exposure days and increased ambulance diversions due to EICU occupancy (adjusted OR, 1.47; 95% CIs, 0.88–2.43) (Table 4).

**Table 4:** Adjusted effects of exposure days on daily ambulance diversions due to EICU occupancy.

Variables	Adjusted OR	95% CI
Exposure days	1.47	0.88–2.43
The number of ambulance acceptances	1.04	0.98–1.11
The presence or absence of rejections due to specialist unavailability	0.97	0.62–1.53
The number of COVID-19 beds occupied	1.18	0.89–1.58
Daily count of patients receiving CRRT	1.12	0.82–1.50
Daily count of patients receiving ECMO	0.99	0.42–2.17
Timeframe (per 3-month period)		
From August 1, 2021, to January 31, 2022	Reference	
From February 1, 2022, to July 31, 2022	1.53	0.71–3.37
From August 1, 2022, to January 31, 2023	3.13	1.49–6.90
From February 1, 2023, to July 31, 2023	2.58	1.28–5.45

Exposure days are defined as any days from the clinical confirmation of brain death in a patient until organ procurement.

*EICU* emergency intensive care unit, *OR* odds ratio, *CI* confidence intervals, *COVID-19* ARS-CoV-2, *CRRT* continuous renal replacement therapy, *ECMO* extracorporeal membrane oxygenation.

## Discussion

This single-center retrospective study revealed that brain-dead donors had longer EICU stays than other patients. The primary analysis showed higher bed occupancy and more ambulance diversions on days with donors’ present (exposure days) compared to other days (control days), even after adjusting for confounders.

A prior meta-analysis examining factors associated with prolonged ICU stay did not address the context of potential organ donors or brain-dead organ donors^21^. Meanwhile, an Australian study noted more ICU admissions for potential donors but found they had shorter ICU stays, suggesting their resource use isn’t disproportionately high^22^. Longer EICU stays for brain-dead donors in our study may result from lengthy discussions on end-of-life care, organ donation consent, and cultural norms around family involvement in medical decisions^7^. This complex process requires time and careful consideration to ensure families are well-consulted and informed decisions are made for quality end-of-life care^23^. Furthermore, in our study, most patients progressing to brain death had post-anoxic encephalopathy, whereas cerebrovascular accidents are more common among brain-dead donors in Spain^4^. This difference likely explains the longer ICU stays in our cohort, as post-anoxic encephalopathy often requires more extended observation and management before brain death diagnosis.

Although multiple factors can affect ICU occupancy rates and define optimal levels, a previous review suggested that optimal ICU occupancy rates should be around 70 to 75%^24^. During our study period, the median EICU occupancy rate was 71%, which is higher compared to the nationwide occupancy rate of around 50% during the COVID-19 pandemic^25^. EICU occupancy was higher on days with brain-dead donors versus control days, suggesting their prolonged stays increase ICU occupancy. While bed types weren’t specified, 21.7% of Japan’s 2021 ambulance diversions were due to full beds for severe cases^26^. Although no direct link was found between diversions and higher mortality^27^, more emergency calls correlated with increased on-scene prehospital times^28^. In fact, more recent study has demonstrated that prolonged on-scene time was linked to increased odds of mortality in trauma patients^29–31^.

Our study highlights the challenge of balancing ICU resources with the need to manage brain-dead donors effectively. High EICU occupancy necessitating ambulance diversions could adversely impact the regional pre-hospital care system by increasing transport times and redirecting patients to alternative facilities, which may lack equivalent critical care capabilities. These diversions can delay definitive care and potentially influence outcomes for critically ill patients. To mitigate these impacts, enhanced regional coordination, hospital collaboration, and policies that optimize ICU resource allocation are essential.

The management of brain-dead donors represents a dual challenge: maximizing organ donation outcomes while maintaining equitable access to ICU resources for all critically ill patients. The prolonged stays associated with brain-dead donors increase ICU occupancy, which may result in ambulance diversions and delays in ICU admission for other patients. Although these practices may indirectly influence patient selection during periods of high occupancy, the prioritization of brain-dead donors reflects their societal importance in addressing the organ shortage crisis.

This study has several limitations. First, it is a single-center, retrospective study with a regional focus, limiting its external validity beyond the Japanese healthcare context. Sensitivity analyses using alternative definitions of exposure days showed no statistically significant associations, likely due to the small sample size, which is a limitation of the study. However, the ICU length of stay reported in our study is consistent with nationwide data^25^, suggesting that this finding is reflective of common practice in Japan. Second, while we examined the impact of EICU occupancy on ambulance diversions, we did not assess the outcomes of patients redirected to other facilities. Future research should address this gap to better understand the broader implications of ambulance diversions. Third, retrospective bias may have influenced the accuracy of our data, potentially misestimating the impact of factors such as ICU length of stay and organ donation decisions. Fourth, while ICU occupancy was a primary factor influencing ambulance diversions, other variables such as emergency department crowding and staffing levels also played a role, complicating the analysis of ICU dynamics and their effects on diversions and outcomes. Finally, our study did not assess whether the presence of brain-dead donors directly influenced ICU admission decisions for other patients. Future research should explore how ICU resource allocation strategies impact patient selection, discharge planning, and long-term patient outcomes.

## Conclusions

Our study found that brain-dead donors had longer EICU stays and increased bed occupancy, leading to more ambulance diversions on exposure days than control days. These findings underscore the critical need for policies that optimize ICU resource allocation while ensuring continued care for brain-dead donors, who play an essential role in addressing the organ shortage crisis. Future research and resource planning must aim to minimize the impact of ambulance diversions on critically ill patients while maintaining the infrastructure necessary to support organ donation programs.

## Supplementary Information

Supplementary Material 1.

## Data Availability

The datasets used and/or analyzed during the current study are available from the corresponding author upon reasonable request.
