# Supplementary material for: Association of emergency intensive care unit occupancy due to brain-dead organ donors with ambulance diversion
Source: Sci Rep. 2025 Apr 12;15:12633. doi: 10.1038/s41598-025-97198-7 (PMC11993566; doi:10.1038/s41598-025-97198-7)
Supplement: Supplementary file 1 — Supplementary Material 1. [file 41598_2025_97198_MOESM1_ESM.docx]

**Supplemental Table 1.** Baseline characteristics of the brain-dead organ donors and other patients admitted to the EICU.

|  | Brain-dead organ donors | Others | *P* value |
| --- | --- | --- | --- |
|  | n=13 | n=1,314 |  |
| Age, median (IQR), y | 43 (25, 57) | 66 (45, 78) | 0.003 |
| Under 18 years old, n (%) | 3 (23.1) | 96 (7.3) | 0.067 |
| Male sex, n (%) | 6 (46.2) | 869 (66.1) | 0.147 |
| Primary disease or injury, n (%) ^a^ |  |  | <0.001 |
| Trauma | 0 | 495 (37.7) |  |
| Sepsis/pneumonia/infectious disease | 0 | 188 (14.3) |  |
| Stroke | 1 (7.7) | 128 (9.7) |  |
| Intoxication | 1 (7.7) | 76 (5.8) |  |
| Neurological | 0 | 73 (5.6) |  |
| Post-cardiac arrest syndrome | 11 (84.6) | 69 (5.3) |  |
| Metabolism disorder | 0 | 65 (4.9) |  |
| Hyperthermia/hypothermia | 0 | 57 (4.3) |  |
| Respiratory | 0 | 36 (2.7) |  |
| Gastrointestinal bleeding | 0 | 28 (2.1) |  |
| Cardiac | 0 | 25 (1.9) |  |
| Burn injury | 0 | 23 (1.8) |  |
| Others | 0 | 51 (3.9) |  |
| APACHE II, median (IQR) ^b^ | 30 (28, 34) | 19 (13, 26) | <0.001 |
| Number of patients who were diagnosed with brain death, n (%) | 13 (100) | 22 (1.7) | <0.001 |
| Number of patients who received CRRT | 2 (15.4) | 62 (4.7) | 0.256 |
| Number of patients who received ECMO | 1 (7.7) | 8 (0.6) | 0.162 |
| ICU length of stay, median (IQR), days | 17 (13, 24) | 2 (1, 6) | <0.001 |
| ICU mortality, n (%) | 13 (100) | 74 (5.6) | <0.001 |

^a^ A patient who had an out-of-hospital cardiac arrest was classified as post-cardiac arrest syndrome regardless of their cause.

^b^ Obtained from patients 18 years old or older.

EICU: emergency intensive care unit, IQR: interquartile ranges, APACHE: acute physiology and chronic health evaluation, CRRT: continuous renal replacement therapy, ECMO: extracorporeal membrane oxygenation, ICU: intensive care unit.
